# Construction and potential application of bacterial superoxide dismutase expressed in *Bacillus subtilis* against mycotoxins

**DOI:** 10.1371/journal.pone.0260047

**Published:** 2021-11-16

**Authors:** Xueqian Dong, Wei Wang, Tianyi Jiang, Yanmin Zhang, Hongyu Han, Yonggang Zhang, Chunyu Yang

**Affiliations:** 1 State Key Laboratory of Microbial Technology, Institute of Microbial Technology, Shandong University, Qingdao, China; 2 Shandong Provincial Key Laboratory of Food & Fermentation Engingeering, Shandong Food Ferment Industry Research & Design Institute, QiLu University of Technology (Shandong Academy of Sciences), Jinan, China; 3 School of Municipal and Environmental Engineering, Shandong Jianzhu University, Jinan, China; Konkuk University, REPUBLIC OF KOREA

## Abstract

Oxidative stress, which could be evoked by numerous inducements including mycotoxins like deoxynivalenol (DON), cause severe damages to organisms. Antioxidants are promising protectants against oxidative stress that could be applied in pharmaceutical, cosmetic, and food and feed industries. In this study, a thermostable and acidophilic superoxide dismutase (*Aa*SOD) was used to develop an antioxidant product that can potentially protect organisms from oxidative stress related damages. The enzyme was successfully expressed as an extracelluar protein in *Bacillus subtilis* with a high yield. To obtain a feasible protocol for industrial production of *Aa*SOD, the fermentation mediums that are commonly used for culturing *B*. *subtilis* were screened, the feasibility of expressing *Aa*SOD without antibiotic as selection pressure was confirmed, and the effect of using lactose as an inducer instead of isopropyl-β-d-thiogalactoside (IPTG) was investigated. Batch fermentation was conducted to validate the optimized conditions for *Aa*SOD production, and 6530 U mL^-1^ of SOD activity was obtained in the fermentation broth. The dry powder product of *Aa*SOD with an activity of 22202 U g^-1^ was prepared by spray-drying and was administrated on zebrafish to test its function as a protectant against DON, and thus gained a significant redress of the reactive oxygen species (ROS) accumulation induced by DON. Taken together, this study provides a feasible protocol to prepare the *Aa*SOD-based antioxidant product that is potentially applied in livestock industry.

## Introduction

Mycotoxins, a structurally diverse group of toxic compounds with mostly small molecular weight, are mainly produced as secondary metabolites by various fungal species under suitable temperature and humidity conditions [1]. Up to now, many mycotoxins have been identified and characterized, and several classes of them received extensive attention, including aflatoxins, trichothecenes, ochratoxins, zearalenone et al. Worldwide,16 million tons of maize, 12 million tons of rice, 1.8 million tons of groundnuts, 378,000 tons of sorghum and millet, 3.7 million tons of copra, and 2.3 million tons of soybeans are contaminated by mycotoxins each year [2]. Such a substantial quantity of food grains contamination further causes considerable livestock losses. Among the mycotoxins, trichothecenes mainly produced by *Fusarium* spp. that had been detected in corn, wheat, barley, and several other crops. As a result, they have been recognized as common contaminants of poultry feeds and feedstuffs [3] and deoxynivalenol (DON or vomitoxin) is one of the most popular trichothecenes. This natural pollutant is particular dangerous due to its broad global distribution and high stability [4].

Like other mycotoxins, DON causes a series of adverse reactions on human or animals, including vomiting, anorexia, extensive necrosis of the oral mucosa and skin, acute effect on the digestive tract, decreased bone marrow, and the disturbance of several other metabolic and immune functions [1, 4]. The underlying mechanisms for these reactions are complex. Oxidative damage is considered as one of the most important factors in the toxicity of DON [5–7]. DON disrupts the normal function of mitochondria and leads to free radicals (mainly reactive oxygen species, ROS) accumulation, which would cause oxidative damage to DNA, lipid, and proteins, impair cell signaling, alter the cells’ antioxidant status, etc., and eventually lead to apoptosis [8, 9].

Due to the critical role of oxidative damage in mycotoxins toxicity and the consequences that it causes, extensive efforts were made to explore potential and effective agents to prevent the oxidative stress and immunotoxicity of DON or other toxins. Various antioxidant agents such as vitamins, quercetin, selenium, glucomannan, polyunsaturated fatty acids, and oligosaccharides, etc., have been reported to have the ability to inhibit trichothecene induced oxidative stress, by suppressing ROS generation, increasing antioxidant enzyme activity, and protecting mitochondria etc. [6, 9–12]. However, some of these agents are low-effective or relatively high-cost to produce, and therefore are unsuitable for application on an industrial scale. As mentioned above, DON could affect antioxidant defense systems, and the manifestations usually include reduced levels of antioxidant enzymes. So, it could be expected that the supplementation of exogenous antioxidant enzymes would be an effective approach to withstand the DON induced oxidative stress of the organism. In livestock and poultry industry, the antioxidant feed additive is widely applied. For example, glucose oxidase, which oxidize β-D-glucose into gluconic acid and produce peroxide with the consumption of oxygen, has been applied as an antioxidant enzyme in feedstuff. This enzyme could reduce oxidative stress by presenting a lower oxygen level, but not by eliminating the oxygen radicals that already formed [13, 14].

Superoxide dismutases (SODs) are a class of metalloenzymes that catalyze the dismutation of superoxide into oxygen and peroxide. As one of the most important antioxidant enzymes in organisms, SODs are prevalent in prokaryotes, archaea, and eukaryotes. These enzymes play a crucial role in the organisms against the oxidative damage by blunting the cascade of oxidations that initiated by superoxide radicals [15]. Compared to non-enzymatic antioxidants, SODs, being catalysts, will not be consumed during the antioxidative reaction, and show high affinity and rate of reaction with ROS. They are considered to be the most potent antioxidants known in the nature, and are widely applied in the pharmaceutical, cosmetic, as well as in the food and feed industries [16]. These properties also endow SODs potentials to be an effective feed additive to withstand the oxidative damages induced by DON and other mycotoxins. However, there are still several practically obstacles need to be addressed. First, high temperature treatment is often used in practical applications while thermal denaturation is an important factor leading to enzyme inactivation. Thermophilic archaea, bacteria and fungi could be potential origins to explore thermotolerant SODs [17–20]. On the other hand, enzyme engineering has also been successfully applied to improve the thermostability of SOD [21]. Second, the acidic tolerance of SOD is also an important property for its application as feed additive, since it has to endure the extremely acidic environment of the stomach. However, in contrast to the abundant resource of thermotolerant SODs, very few SOD has been reported to be active or stable under extremely acidic conditions [17, 22]. Third, a low-cost and high-efficient production procedure should be established for its large-scale applications [16].

Concerning the demands and problems addressed above, we expect to explore a novel feed additive of SOD, to reduce the damages caused by DON and other mycotoxins. In our previous study, we have identified a SOD from *Alicyclobacillus* (*Aa*SOD). This enzyme is perfectly ideal to meet the requirement as a feed additive, including the highest activity in the mesophilic or neutral environments, especially high stabilities under high temperatures and extremely acidic environments [17]. In this study, an engineered *Bacillus subtilis* strain expressing extracellular *Aa*SOD was constructed to achieve low-cost and high-efficient production of this enzyme. Fermentation process was optimized and high extracellular SOD activity was obtained. The SOD product was further applied on zebrafish to assess its functions in reducing the DON-induced oxidative stress.

## Materials and methods

### Ethics statement

All the experimental protocols about zebrafish in this study were approved by the Animal Care and Use Committee of Biology Institute of Shandong Academy of Sciences (protocol number SWS20210802).

### Reagents

Yeast extract and tryptone were purchased from Oxoid Ltd. (England). Coomassie brilliant blue R250, G250, and materials for gel electrophoresis were obtained from Sigma Chemical (St Louis, Missouri). Endonucleases and DNA polymerase were obtained from Takara (Japan). The T5 exonuclease-dependent DNA assembly reaction mixture was a gift from Professor Gu Lichuan (Shandong University). All other reagents were of AR grade.

### Strains, plasmids, and media

Strain *Alicyclobacillus* sp. HJ, in which the *Aa*SOD was identified, was isolated from the Tengchong hot spring [23]. The clone vector pEASY-blunt and *Escherichia coli* DH5α was used for gene cloning and fidelity confirmation. The recombinant *B*. *subtilis* expression vector pHT43 carrying *Aa*SOD encoding gene was transformed into *B*. *subtilis* SCK6 to construct the engineered strain [24].

Luria-Bertani (LB) medium was used for routine cultivation of stains. For the expression of *Aa*SOD, 0.2 mM Mn^2+^ was added to LB medium. Fermentation medium 1 (FM1) of recombinant *B*. *subtilis* contains (L^-1^) 10 g glucose, 10 g beef extract, 10 g tryptone, 5 g yeast extract, 5 g NaCl, and 0.03 g MnSO_4_. Fermentation medium 2 (FM2) contains (L^-1^) 20 g glucose, 20 g tryptone, 10 g yeast extract, and 0.03 g MnSO_4_. Fermentation medium 3 (FM3) contains (L^-1^) 20 g glucose, 4 g corn steep liquor, 5 g yeast extract, 2 g NaCl, and 0.03 g MnSO_4_. Fermentation medium 4 (FM4) contains (L^-1^) 10 g glucose, 20 g corn flour, 4 g corn steep liquor, 10 g yeast extract, 2 g NaCl, and 0.03 g MnSO_4_. For antibiotic selective media, ampicillin or chloramphenicol was added at 100 μg mL^-1^ or 5 μg mL^-1^, respectively.

### Construction of *Aa*SOD over-expressing *B*. *subtilis* strain

The construction of recombinant plasmid pHT43-*AaSOD* for the expression of *Aa*SOD was carried out following the T5 exonuclease-dependent assembly protocol [25]. The *Aa*SOD gene was amplified using genomic DNA of *Alicyclobacillus* sp. HJ as template, and the primers used are *Aa*pHT-F (5’–ACAAAAACATCAGCCGGATCCATGCCACATCAACTCCCACC–3’) and *Aa*pHT-R (5’–CTGCGACGTCTTAGTGATGGTGATGGTGATGGCCGTTCAGCGCGGCCTC–3’), which contain restriction sites *BamH*I and *Aat*II (underlined) and homologous arms for DNA fragments assembly, respectively. Also, a 6×His tag sequence was introduced into the *C*-terminus of *Aa*SOD by adding its encoding sequence in the downstream primer. The pHT43 plasmid was also amplified using primers pHT*Aa*-F (5’–CATCACCATCACCATCACTAAGACGTCGCAGCCCGCCTAATG–3’) and pHT*Aa* -R (5’–GGGAGTTGATGTGGCATGGATCCGGCTGATGTTTTTGTAATCG–3’) that contain homologous arms. The PCR products were then mixed, treated by T5 exonuclease-dependent DNA assembly reaction mixture, and then transformed into competent cells of *E*. *coli* DH5α. The correct clones were picked, and further verified by double digestion and sequencing of recombinant plasmid.

Preparation and transformation of competent cells of *B*. *subtilis* SCK6 was carried out following the protocol reported previously [24]. The positive colonies were selected using chloramphenicol resistance as an indicator, and were further verified by colony PCR and sequencing.

### Protein expression and purification

Cells of *B*. *subtilis* harboring pHT43-*Aasod* were cultured in 5 mL LB medium at 37 °C for 12 h. Subsequently, the culture was inoculated into 100 mL LB medium (with 1 mM Mn^2+^) at a ratio of 1:100 and cultured at 37 °C. When the OD_600nm_ value of the culture reached to 0.5, isopropyl-β-D-thiogalactoside (IPTG) was added into a final concentration of 0.5 mM for protein induction. After cultivation at 37 °C, 200 rpm for 18 h, the culture was centrifuged at 12,000 × g for 10 min, and the supernatant was collected for protein purification. Ammonium sulfate was added to the supernatant to a final concentration of 75% (m/v) to precipitate the extracellular proteins. The solution was centrifuged at 12,000 × g for 10 min, and the sediment was then resuspended in buffer A (20 mM Tris–HCl, 0.5 M sodium chloride, pH 8.0). Dialysis desalination was performed to remove the ammonium sulfate. The desalted solution was applied to the HisTrap crude column and the target protein was eluted with buffer B (20 mM Tris–HCl, 0.5 M sodium chloride, 250 mM imidazole, pH 8.0). The expression level and purity of target protein were verified by SDS-PAGE (12% w/v) using a Bio-Rad Mini-PROTEAN TETRA electrophores system (CA, USA).

### Comparison of fermentation mediums

Cells of *B*. *subtilis* harboring pHT43-*Aasod* were cultured in LB medium at 37 °C for 12 h. Subsequently, the culture was inoculated into 100 mL LB (with 0.2 mM extra Mn^2+^ added), FM1, FM2, FM3, or FM4 medium at an amount of 5% (v/v) and cultured at 37 °C, 200 rpm for 24 h. The supernatant of each culture was collected for SOD activity assay. The medium with a maximum activity was obtained and chosen for the following study.

### Optimization of lactose induction

Lactose was tested as the substitution of IPTG for protein expression induction in the fermentation processes. The amount of lactose, time interval between inoculation and induction, and cultivation time after induction were optimized following an L_9_-orthogonal array. The orthogonal array was designed and analyzed using MINITAB 13.30 software. Table 1 depicted the detail conditions and the L_9_-orthogonal array applied in this study.

**Table 1:** L_9_-orthogonal array for lactose induction conditions.

Run	Components			Induction conditions			SOD activity^a^ (U/mL)
	A	B	C	A	B	C	
**1**	1	1	1	4	2	24	433 ± 14
**2**	1	2	2	4	4	36	666 ± 33
**3**	1	3	3	4	6	48	1073 ± 94
**4**	2	1	2	6	2	36	805 ± 22
**5**	2	2	3	6	4	48	1496 ± 34
**6**	2	3	1	6	6	24	526 ± 12
**7**	3	1	3	8	2	48	1332 ± 35
**8**	3	2	1	8	4	24	501 ± 11
**9**	3	3	2	8	6	36	1055 ± 55

Where: A = time interval between inoculation and induction (h), B = lactose concentration (g L^-1^), C = cultivation time after induction (h).

^a^ Results are mean ± SD of three determinations.

### Batch fermentation in stirred bioreactors

Batch fermentation was conducted in a 10 L stirred bioreactor (Bailun Biological Technology, China) with 6 L of initial medium. The fermentation was initiated by inoculation with a seed culture (10% v/v), and carried out at 37°C. The initial pH was adjusted to 7.0 and not controlled during the fermentation process. The aeration rate was 1.0 vvm. The agitation speed was initially 200 rpm, and was elevated to 400 rpm at the exponential growth phase (6 h). Lactose was added according to the optimized condition. Samples were collected every 4 h to determine the biomass and SOD activity. Glucose was measured by SBA-40C glucose biosensor (Biology Institute of Shandong Academy of Sciences, China), and reducing sugar was measured by Fehlings reagent titration.

### Preparation of dry powder of fermentation broth by spray drying

The fermentation broth was centrifuged at 8,000 × g for 10 min to remove the cells. Ingredients including CaCO_3_ and mycose were added into the supernatant at amounts of 25% (m/v) and 1% (m/v), respectively. The mixture was spray dried with a BUCHI Mini Spray Dryer B-290 (BUCHI Labortechnik AG, Switzerland). The inlet flow with peristaltic pump is 15‒40 mL min^-1^, the spray pressure is 5–6 bar, and the inlet temperature is 120–160°C.

### SOD activity assay

Following the manufacture’s instruction, the SOD activity was measured using a SOD Assay Kit (Dojindo Laboratories, Japan) based on water-soluble tetrazolium (WST-1) method [26].

### Administration of *Aa*SOD on zebrafish against DON

Zebrafish (*Danio rerio*) of the wild-type AB strain were used in this study. A total of 60 zebrafish of 3 dpf were randomly assigned to 5 groups with 12 zebrafish per group. In the control group, zebrafish were cultured normally. In the DON group, the DON toxin was dissolved in methanol and diluted to 3 ppm in the culture water. In the 3 groups that supplied with *Aa*SOD, besides 3 ppm DON, the SOD product was also added with three different SOD doses (0.25, 0.5, or 1.5 U mL^-1^) in the culture water. Zebrafish of all the groups were kept under a 14/10 h light/dark cycle at 28 ± 0.5°C in a closed flow-through system. All experiments followed the protocols outlined by the Engineering Research Center of Zebrafish Models for Human Diseases and Drug Screening of Shandong Province.

### Measurement of ROS generation

The fluorescent probe dye 2′,7′-dichlorofluorescein diacetate (DCFH-DA) purchased from Nanjing Jiancheng Bioengineering Institute (Nanjing, China) was used to determine the generation of ROS. After 3 days of culture, 8 AB strain zebrafish were randomly selected from each group, and incubated with 20 μM DCFH-DA for 1 h in the dark at 28°C. Then, they were washed twice with culture water and imaged using a fluorescence microscope (Olympus). Image-Pro Plus software was used to quantify the fluorescence intensity.

### Statistical analysis

All experiments were performed at least in triplicate. Data are expressed as the mean ± SD. Statistical comparisons were made between two groups with the *t*-test and between multiple groups by ANOVA. *p* < 0.05 was considered as significant.

## Results and discussion

### Expression of *Aa*SOD in *B*. *subtilis*

**Fig 1:**
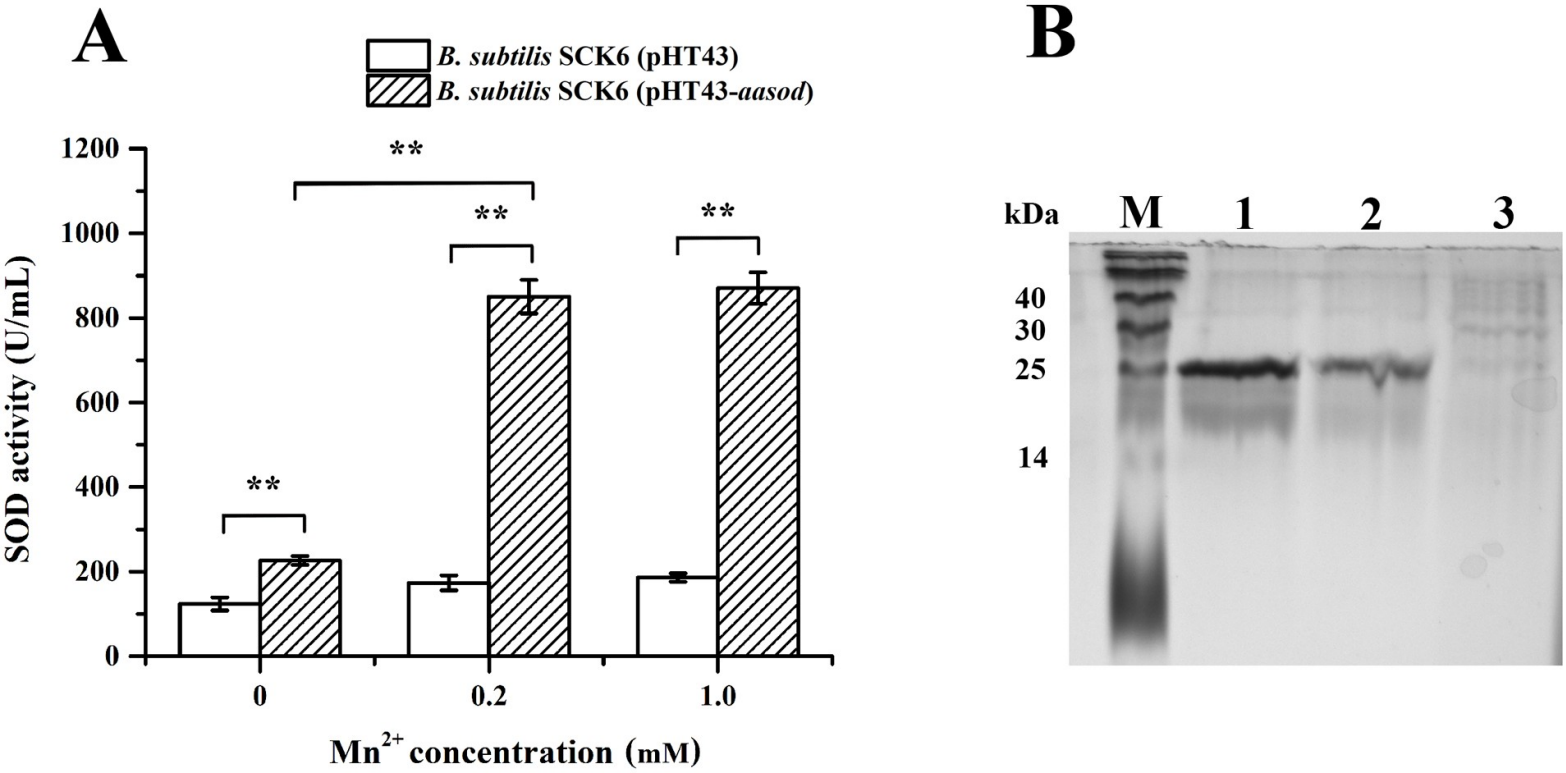
Heterologous expression of *Aa*SOD in *B*. *subtilis*. (A) Confirmation of the Mn^2+^-dependent extracellular SOD activity of recombinant *B*. *subtilis* SCK6 strain harboring pHT43-*Aasod* plasmid. The data are expressed as the mean ± SD of three independent experiments. **Represents significant difference (*p* < 0.01) between groups. (B) SDS-PAGE analysis of the purified recombinant *Aa*SOD. Lane M, molecular weight markers; lane 1, 50% buffer A+50% buffer B eluted sample obtained from HisTrap chromatography with *B*. *subtilis* SCK6 (pHT43-*Aasod*); lane 2, 100% buffer B eluted sample obtained from HisTrap chromatography with *B*. *subtilis* SCK6 (pHT43-*Aasod*); lane 3, 50% buffer A+50% buffer B eluted sample obtained from HisTrap chromatography with *B*. *subtilis* SCK6 (pHT43).

In this study, *B*. *subtilis* was used as a host in the exogenous expression of *Aa*SOD, for its advantages of safety and diversified expression-secretion system [27, 28]. *Aa*SOD was expressed in *B*. *subtilis* with a signal peptide of α-amylase (SP-amyQ) at *N*-terminus (included in the pHT43 vector) and a 6×His tag at *C*-terminus. Since the *Aa*SOD has been characterized to be a Fe/Mn type SOD, with the Mn^2+^-binding being much more effective on the activity than Fe^2+^ [17], Mn^2+^ was added to the fermentation medium. As shown in Fig 1A, when compared with the control strain (*B*. *subtilis* SCK6 harboring empty vector), obvious SOD activity could be detected in the supernatant of culture medium after 24 h of induction by IPTG. Similar to the results obtained using *E*. *coli* as the host strain [17], the Mn^2+^ enriched medium provides much higher activity (850 ± 40 U mL^-1^, with 0.2 mM Mn^2+^) than the natural medium (226 ± 11 U mL^-1^). However, the medium with 1.0 mM Mn^2+^ (870 ± 37 U mL^-1^) did not show significant difference compare to that with 0.2 mM Mn^2+^, which means 0.2 mM Mn^2+^ was sufficient for heterologous expression of *Aa*SOD. After purification of the culture supernatant by a His-Trap affinity column, a band of approximately 25 kDa was detected (Fig 1B). This is in accordance with the theoretical molecular weight of *Aa*SOD (22.82 kDa) and confirmed the extracellular expression of *Aa*SOD. The yield of protein was estimated to be 87.5 mg L^-1^, which was comparable to the yield by using *E*. *coli* for intracellular expression in our previous study [17]. Our results show that it is feasible to use *B*. *subtilis* as a host for SOD expression, which could facilitate its application in the fields that concerning biological safety.

### Comparison of media for SOD fermentation

*B*. *subtilis* is a strain with various applications, and the medium used for its fermentation varied according to the field that it was applied. With aims to develop an antioxidant product that is suitable to be applied in livestock and poultry industry, it is necessary to find a low-cost and high-efficient fermentation medium for production of *Aa*SOD. A screening of media with five candidates, including LB medium as a control, was carried out in this study. All of these media (listed in Materials and methods section) have been widely applied for the culture of *B*. *subtilis* in different filed, and they differ in carbon source, nitrogen source, mineral salt, or trace elements. In addition, Mn^2+^ of 0.2 mM was added to each medium to meet the requirement of *Aa*SOD. However, since a lot of solid content remain in the media FM3 and FM4, it is impossible to control the time of IPTG induction according to the OD_600nm_ of culture as routine protocols. Hence, an identical induction time of 4 h after inoculation, which is approximately the proper induction time for LB medium, was applied for all the five media.

**Fig 2:**
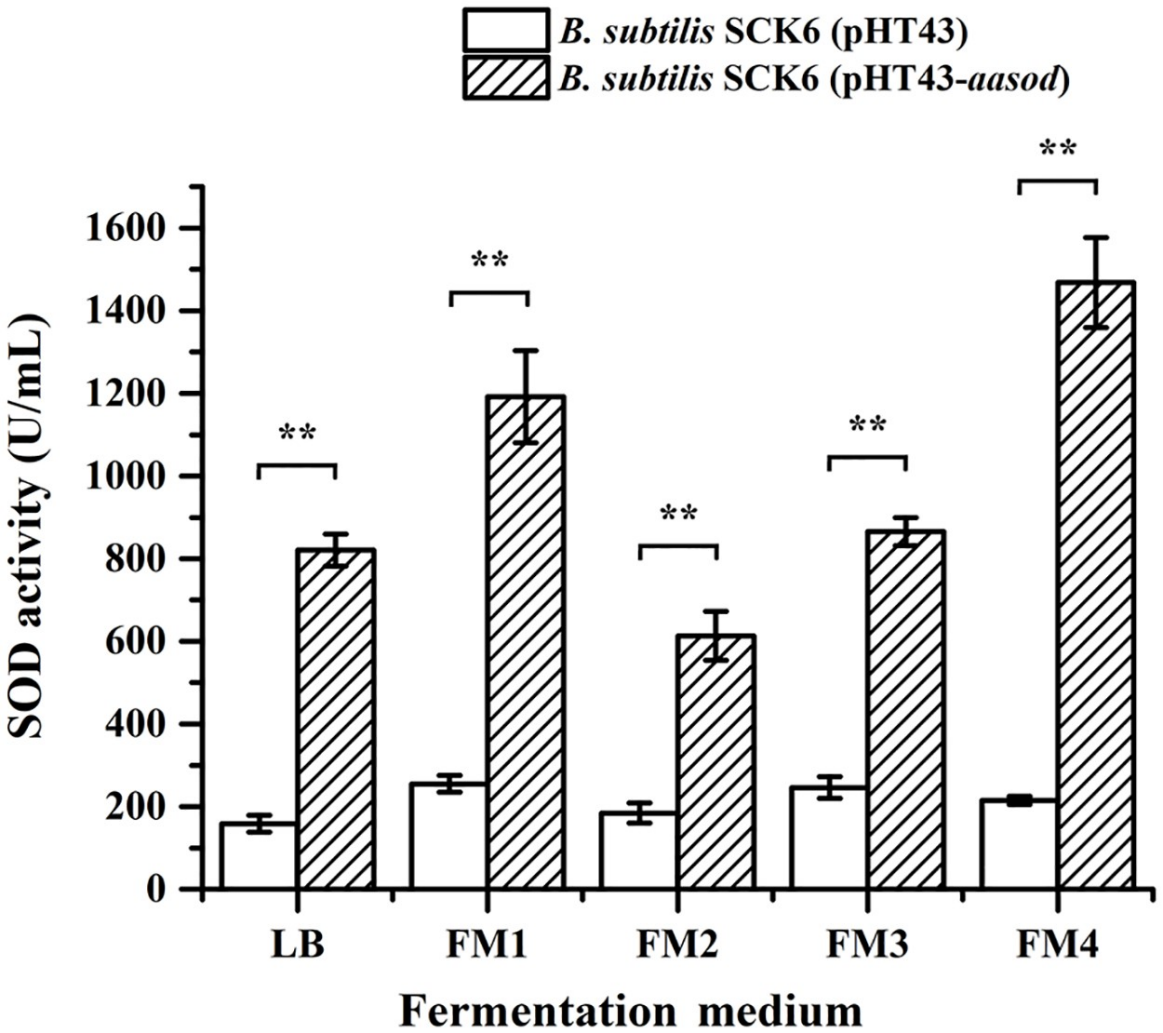
Comparison of fermentation media for *Aa*SOD production. The data are expressed as the mean ± SD of three independent experiments. **Represents significant difference (*p* < 0.01) between groups.

As shown in Fig 2, after 24 h of fermentation, the highest SOD activity was obtained in medium FM4 (1468 ± 109 U mL^-1^), which was 1.8 folds higher than that was obtained with Mn^2+^ enriched LB medium. The medium FM2 gave the lowest SOD activity, which was only 613 ± 59 U mL^-1^. The result shows that the expression efficiency of *Aa*SOD by *B*. *subtilis* varies dramatically according to the media applied. Nevertheless, this is a promising result since medium FM4 originally is a widely applied medium for production of *B*. *subtilis* spores for agriculture and feed use, and so it is not conflicting between the production of *Aa*SOD and its original application. Furthermore, by using corn starch as the main carbon and soya bean meal as the nitrogen source, this medium has the advantages of low cost and thus is beneficial to the industrial production.

### Segregational plasmid stability

It is well-known that the stability of segregational plasmid have a significant influence on the production of heterologous proteins or plasmids themselves [29]. For the expression system used in this study, chloramphenicol resistance is applied in *B*. *subtilis* expression vector pHT43 and used as a marker to ensure the partition of plasmids on to the daughter cells. However, the costs of adding antibiotics could be an adverse factor for an industrial production, and more importantly, antibiotic is not applicable in all pharmaceutical and food production processes. To ensure the feasibility of applying our product as a feed additive, antibiotic should be excluded in the fermentation medium. To assess the segregational stability of recombinant pHT43 plasmid, the engineered strain was cultivated in FM4 medium without chloramphenicol for 36 h (with IPTG added at 4 h), and the final culture was diluted for colony counting on plates of LB agar medium with or without chloramphenicol addition. With a plasmid retention rate of 39.1%, 91 ± 8 or 36 ± 5 colonies was obtained on the agar medium without or with chloramphenicol, respectively. Similar experiments were carried out using FM4 medium with chloramphenicol addition, and the plasmid retention rate was 87.2% (102 ± 4 colonies on the regular agar plate and 89 ± 3 colonies on the antibiotic agar plate). This result indicates that the recombinant pHT43 plasmid had an apparent loss, which conforms to the consensus that as a metabolic burden for the cells, plasmids are preferred to be abandoned by cells during cultivations [29, 30]. Interesting, the SOD activities in the tested two conditions did not vary as significantly as the plasmid retention rates. Almost the same activities were obtained using medium with or without chloramphenicol (1502 ± 127 U mL^-1^ for chloramphenicol-added medium and 1428 ± 52 U mL^-1^ for chloramphenicol-free medium). The underlying reason of these results needs to be addressed in further studies. Nevertheless, these results guarantee that the antibiotic need not to be added into the medium for a practical fermentation process, as long as ensuring the stability of plasmid in the preservation of recombinant strain by using chloramphenicol as the selection pressure.

### Optimization of lactose induction conditions

Although high SOD activity could be obtained using IPTG as an inducer, this chemical is relatively expensive and thus increase the cost of practical production. Furthermore, the presence of IPTG as a contaminant of the final product is problematic. As an alternative, lactose could also be used as an inducer for lac operon. As a completely non-toxic chemical, lactose is readily available in large quantities as a by-product of the dairy industry and so it is much economic than IPTG. However, the conditions using lactose as the inducer must be optimized due to the physiological response of the cell to the presence of sugar and the fact that lactose is metabolized by the cell [31]. In this study, the amount of lactose, time interval between inoculation and induction, and cultivation time after induction were optimized using an L_9_-orthogonal array method.

Table 2 represents the response table for means of SOD activities and signal to noise rations obtained with L_9_-orthogonal array. A higher delta value indicates greater effect of that component. The result suggests that the cultivation time after induction had a major effect and amount of lactose had the least effect on SOD activity. The optimal levels for the amount of lactose, time interval between inoculation and induction, and cultivation time after induction were 4 g L^-1^, 6 h, and 48 h, respectively. It is obvious that longer cultivation time enhanced the final SOD activity. It could be speculated that this benefits from the high stability of *Aa*SOD in extracellular conditions and so it could accumulate continuously. Furthermore, the long period needed for lactose induction may resulted from the presence of glucose and corn starch in the medium which would delay the fully induction of lac operon. The lactose permease transport lactose into the cell, and then the β-galactosidase convert lactose to allolactose, which is the actual inducer of lac operators and will not be fully activated during the early period of induction [32]. Besides lactose induction, the recombination strain was also induced with IPTG for comparison. After 48 h induction, the highest SOD activity was obtained (2384 ± 113 U mL^-1^), which is only 37.2% higher than that of lactose induction (1496 ± 34 U mL^-1^). Overall, for practical production, the advantages of lactose over IPTG in terms of cost and safety make it a feasible choice for production of pharmaceutical and food products.

**Table 2:** Response table for means and S/N ratio.

Levels	A		B		C	
	Mean	*S*/*N*	Mean	*S*/*N*	Mean	*S*/*N*
**1**	724	25.6	857	30.8	486	31.8
**2**	942	32.3	888	30.6	842	27.6
**3**	962	30.1	885	26.5	1300	28.6
**Delta**	238	6.6	31	4.3	814	4.2
**Rank**	2		3		1	

Where: A = time interval between inoculation and induction (h), B = lactose concentration (g L^-1^), C = cultivation time after induction (h).

### Batch fermentation and product preparation

**Fig 3:**
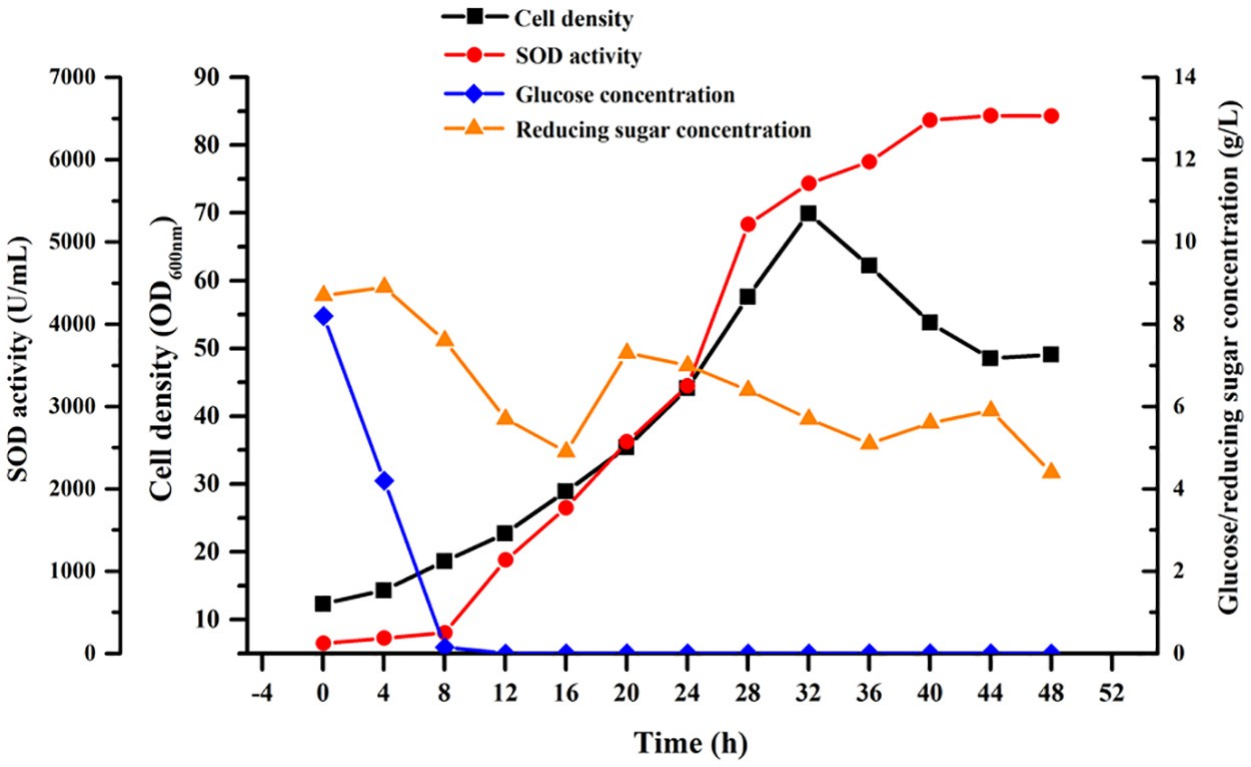
Time courses of cell growth, sugar concentrations, and SOD activity in batch fermentation by recombinant *B*. *subtilis* expressing *Aa*SOD in a 10 L stirred bioreactor.

Batch fermentation was carried out in a 10 L stirred fermenter, by using the chloramphenicol-free FM4 medium as the fermentation medium. LB medium with chloramphenicol was used as primary strain activation, and FM4 medium without chloramphenicol was used as secondary seed activation and fermenter production to ensure only trace antibiotic residue remained in the final broth. Lactose was added into the culture after 6 h of inoculation to a final concentration of 4 g L^-1^. As shown in Fig 3, the glucose which served as rapidly available carbon source was exhausted after 8 h, while the concentration of reducing sugar fluctuated continuously due to the presence of corn starch. The SOD activity increased continually and reached to a maximum activity of 6530 U mL^-1^ until 44 h cultivation, which is much higher than that of shake flasks. We suspect it was because the remarkably improved dissolved oxygen and mass transfer of stirred fermenter promoted cell growth and protein expression.

The dry powder of final fermentation broth was prepared by spray drying, with CaCO_3_ supplementation to increase solid content (25%, m/v) and 1% mycose as the enzyme protectant. The SOD activity of product was 22202 U g^-1^, with a total recovery rate of 91.2%. The loss of enzyme activity may be partly caused by some residual in the spray drying system, and also because of the slight damage of enzyme by the extreme high temperature during the process. Some other technics, like freeze drying, would provide a better protection of extracellular *Aa*SOD. However, the relatively high costs of such technics would restrict their applications in preparing powder additives for stockbreeding. Based on these results, it is feasible for the fermentation production of *Aa*SOD in an industrial scale. To our best knowledge, the most commonly production strategies for commercially available SOD products are extracting from animals, plants, or marine materials [33], which would be limited by their unstable material resources, relatively low contents, and limited extraction yields. Comparatively, microbial fermentation-based strategies for proteins production are more cost-effective since microbes could be cultivated easily at large scale. Some microbial hosts, such as *E*. *coli* and yeast, have been reported to be utilized for the production of recombinant SODs [34, 35]. However, *B*. *subtilis*, as another widely applied host for recombinant protein expression, has not yet been used. Indeed, using *B*. *subtilis* strains as hosts could further lower the cost of SOD production since they could achieve robust growth with crude and low-cost materials in the fermentation medium, such as those used in this study. Once this protocol is scaled up, the unit material cost could remain stable, while the unit energy consumption would be further reduced, since the widely approved fact that larger bioreactors provide lower unit energy consumptions. However, challenges are still need to be faced for production of *Aa*SOD in an industrial scale. For examples, the high oxygen consumption of *B*. *subtilis* cells during robust growth need to be satisfied by the bioreactors, and the loss of SOD activity might be amplified when an industrial-scale spray dryer is applied since it is operated at higher temperature and longer period.

### Application of *Aa*SOD product on zebrafish against DON

**Fig 4:**
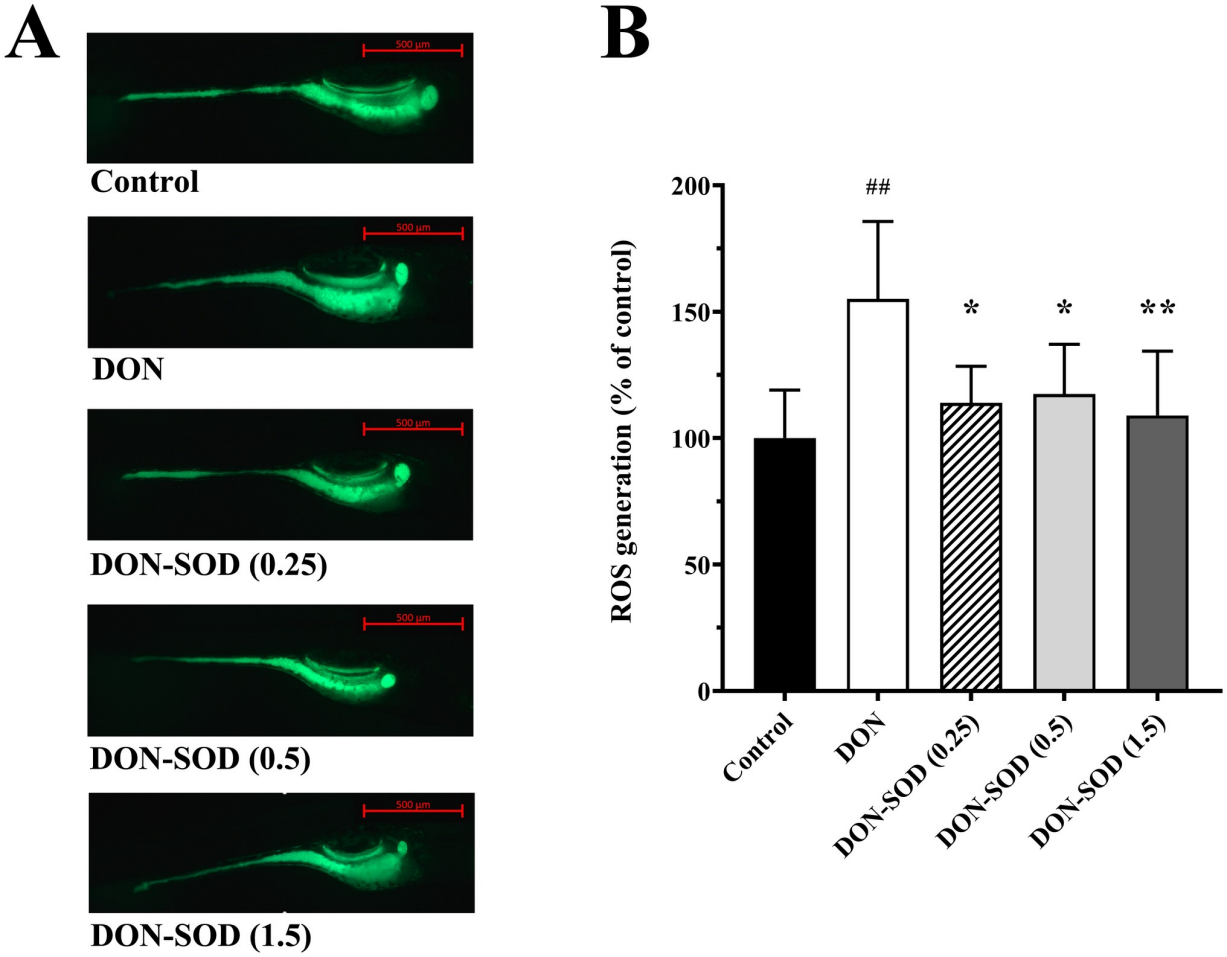
Effects of DON and *Aa*SOD product on ROS accumulation in zebrafish. (A) Fluorescence images of ROS generation in zebrafish of different groups. In the control group, zebrafish were cultured without adding DON or *Aa*SOD. For each group except the control group, DON was added to the culture water to a concentration of 3 ppm. *Aa*SOD was not supplied in the DON group, while was supplied at concentrations of 0.25 U mL^-1^, 0.5 U mL^-1^, or 1.5 U mL^-1^ in the DON-SOD (0.25) group, DON-SOD (0.5) group, or DON-SOD (1.5) group. (B) The quantified fluorescence intensities of zebrafish in different groups. The IOD values analyzed by Image-Pro Plus software were adjust using the mean value of control group as 100%. The data are expressed as the mean ± SD of eight zebrafish. The significance of each DON-added group vs control group was analyzed with *t*-test, and ^##^represents *p* < 0.01. The significance among the 4 DON-added groups were analyzed with ANOVA, and *represents *p* < 0.05 and ** represents *p* < 0.01 vs the DON group.

The *in vivo* effect of the *Aa*SOD product against oxidative stress caused by mycotoxins was further investigated. Zebrafish (*Danio rerio*), which has been extensively studied and well described for environmental toxicity studies [36], were used as an animal model. As a frequent contaminant of food and has specific correlation between toxicological effects and oxidative damages, DON was used as the representative mycotoxin. As introduced above, ROS accumulation could serve as a sensitive biomarker of oxidative damage caused by DON in experimental animals. So, the ROS accumulations of zebrafish that not treated, treated with DON, or with DON and *Aa*SOD were simultaneously assessed by DCFH-DA staining (Fig 4A), and integral optical density (IOD) were applied to quantify the fluorescence intensities (Fig 4B). The results showed that the zebrafish treated with DON possessed higher fluorescence than that of the control group (*p* < 0.01). Regarding the mean fluorescence intensity value of control group as 100, the relative value of DON group raised to 155.1 ± 30.6, indicating that DON exposure evokes ROS generation. The fluorescence intensities were redressed in different degrees by the supplement of *Aa*SOD. The group supplied with 0.25 U mL^-1^, 0.5 U mL^-1^, or 0.5 U mL^-1^ *Aa*SOD yielded a relative fluorescence intensity of 113.9 ± 14.5, 117.4 ± 19.7, or 109.0 ± 25.4, respectively. All the three groups did not show significant difference in ROS accumulations when compared to the control group, while decreased significantly than the DON treated group (*p* < 0.05 for the group supplied with 0.25 or 0.5 U mL^-1^ *Aa*SOD, and *p* < 0.01 for the group supplied with 1.5 U mL^-1^ *Aa*SOD). Thus, considering the cost and effect comprehensively, 0.25 U mL^-1^ should be the suggested dose of *Aa*SOD for this example case. These results show that the product developed in this study could be an effective protector of animals against oxidative damage of mycotoxins such like DON, although the optimum dose need to be ascertained specifically according to animals that the enzyme was applied to. As important signaling molecules, ROS of low concentration play important roles in host defense systems in a variety of organisms [37]. However, the ROS toxicities have been widely recognized when there is an imbalance between production of ROS and cellular antioxidant defenses. By causing damages to proteins, lipids, and DNA, ROS accumulation participates in stimulating cell injury, physiological dysfunction, and various diseases [38, 39]. As a lot of chemicals could induce apoptosis and cytotoxicity through increasing ROS production and oxidative stress [38, 40, 41], it could be expected that this *Aa*SOD product, serving as an antioxidant that eliminating ROS, could also protect animals from damages of other toxic chemicals besides mycotoxins.

## Conclusions

The aim of this study is to develop a potential antioxidant enzyme product (*Aa*SOD) that is suitable for oral administration of animals, by releasing the oxidative damages from the mycotoxins such as DON. As a thermostable and acidophilic protein identified from *Alicyclobacillus* sp. HJ, *Aa*SOD was successfully extracellularly expressed in *B*. *subtilis*. The excellent properties of *Aa*SOD and high expression efficiency endow it great potential for practical application. The practical feasibility of this product were addressed in the following issues. Firstly, the medium used for production of *B*. *subtilis* spores was found to be suitable for expression of *Aa*SOD. Secondly, the chloramphenicol, which was essential for the construction and stability of the engineered plasmid in *B*. *subtilis* strain, was not needed in the production duration. Thirdly, for product safety of oral administration, lactose was confirmed as an economic and alternative inducer for *Aa*SOD production. Fourthly, benefit from the high thermostability of *Aa*SOD, the product prepared by spray-drying contains 22202 U g^-1^ of SOD activity with a high recovery rate of 91.2%. Furthermore, this product was demonstrated to be an effective antioxidant against DON, by remarkably redressing the ROS accumulation of the zebrafish. Overall, the SOD product developed in this study could counteract the damages caused by DON or other toxic chemicals in an antioxidative way, and thereby will be a promising economic and effective antioxidant feed additive for livestock industry.

## Supporting information

S1 Raw images (PDF)
