# Supplementary material for: Construction and potential application of bacterial superoxide dismutase expressed in Bacillus subtilis against mycotoxins
Source: PLoS One. 2021 Nov 16;16(11):e0260047. doi: 10.1371/journal.pone.0260047 (PMC8594817; doi:10.1371/journal.pone.0260047)
Supplement: S1 Raw images — (PDF) [file pone.0260047.s001.pdf]

Fig 1B

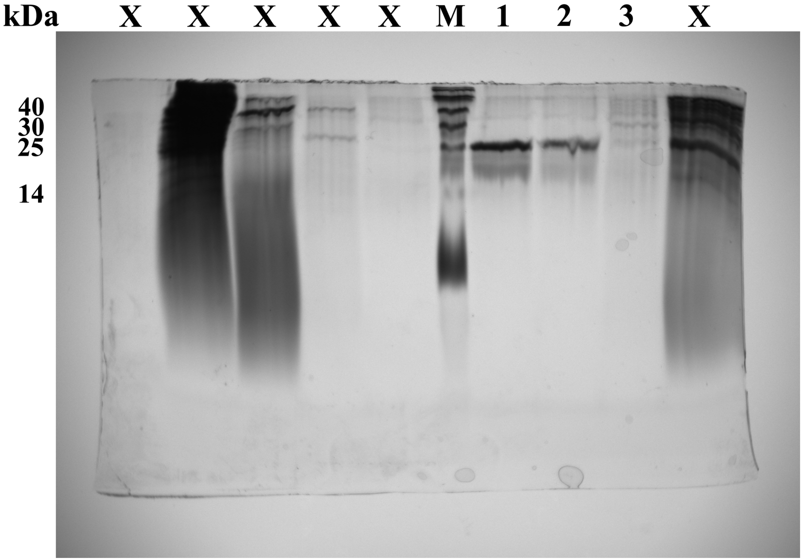

M: molecular weight markers.

1: 50 % buffer A+50 % buffer B eluted sample obtained from HisTrap chromatography with *B. subtilis* SCK6 (pHT43-*Aasod*).

2: 100 % buffer B eluted sample obtained from HisTrap chromatography with *B. subtilis* SCK6 (pHT43-*Aasod*).

3: 50 % buffer A+50 % buffer B eluted sample obtained from HisTrap chromatography with *B. subtilis* SCK6 (pHT43).

X: not included in the final figure.

The image was captured by a GenoSens 2000 Series Gel Doc System (Clinx Science Instruments, China).
